# Role of Thromboelastometry in Sepsis-Induced Coagulopathy: A Narrative Review

**DOI:** 10.3390/medsci14030385

**Published:** 2026-07-09

**Authors:** Andrzej Lubiewski, Mariusz Siemiński

**Affiliations:** Department of Emergency Medicine, Medical University of Gdansk, 80-210 Gdansk, Poland; sieminski@gumed.edu.pl

**Keywords:** sepsis-induced coagulopathy (SIC), sepsis, rotational thromboelastometry (ROTEM), disseminated intravascular coagulation (DIC), immunothrombosis, fibrinolysis shutdown

## Abstract

Sepsis-induced coagulopathy (SIC) represents an early, predominantly prothrombotic disturbance that is difficult to detect using conventional coagulation tests. This review aims to evaluate whether rotational thromboelastometry (ROTEM) provides added value in the diagnosis and phenotyping of SIC compared with standard laboratory parameters. We summarize current evidence on SIC pathophysiology and its relationship to disseminated intravascular coagulation (DIC), with particular focus on mechanisms driving hypercoagulability and fibrinolytic shutdown. The limitations of routine tests, platelet count, PT/INR, fibrinogen, and fibrin-related markers are examined, especially their inability to capture early dynamic changes in coagulation. We then outline the principles of ROTEM and review clinical studies assessing its application in sepsis. Available data consistently demonstrate increased clot firmness, frequently driven by elevated fibrinogen, along with impaired fibrinolysis. These patterns often precede overt SIC or DIC and are associated with worse clinical outcomes, including organ dysfunction and mortality. Finally, we discuss the implications of these findings for clinical management. While routine anticoagulation in unselected septic patients remains unsupported, integrating SIC criteria with ROTEM profiles may improve risk stratification and help identify prothrombotic phenotypes that could benefit from targeted therapies. Overall, ROTEM appears to be a valuable adjunct to conventional testing, with potential to refine diagnostic accuracy and guide future interventional strategies in septic coagulopathy.

## 1. Introduction

Sepsis is defined as a life-threatening organ dysfunction caused by a dysregulated host response to infection [1,2]. Beyond the classical paradigm of overwhelming inflammation, sepsis is now understood as a complex syndrome involving tightly interconnected immune, endothelial, and haemostatic pathways that collectively contribute to microvascular dysfunction and organ failure [1,3]. Among these processes, activation of the coagulation system plays a central role, linking inflammation with thrombosis in a phenomenon often referred to as thromboinflammation.

Sepsis-induced coagulopathy (SIC) represents an early and clinically relevant manifestation of this dysregulated haemostatic response. It is characterized predominantly by a prothrombotic state driven by increased thrombin generation, suppression of endogenous anticoagulant pathways, and impaired fibrinolysis [4,5,6]. Importantly, SIC is not synonymous with overt disseminated intravascular coagulation (DIC), but rather reflects an intermediate stage along a continuum that may progress to consumptive coagulopathy in more advanced disease [6,7]. Recognition of SIC has important clinical implications, as it identifies a subgroup of septic patients at increased risk of organ dysfunction and mortality, often in the absence of significant bleeding [5,8,9].

To facilitate early identification, the International Society on Thrombosis and Haemostasis (ISTH) introduced a simplified SIC scoring system incorporating platelet count, prothrombin time (PT/INR), and the Sequential Organ Failure Assessment (SOFA) score [3,10]. While this approach improves clinical applicability and risk stratification, it remains dependent on conventional coagulation assays that provide only a static and partial representation of haemostatic function [3,4]. These limitations are particularly relevant in sepsis, where coagulation is highly dynamic and influenced by cellular components, endothelial activation, and evolving fibrinolytic activity.

In this context, viscoelastic testing methods such as rotational thromboelastometry (ROTEM) have gained increasing attention. Unlike conventional assays, ROTEM provides a real-time, whole-blood assessment of clot initiation, propagation, strength, and breakdown, thereby offering a more integrated evaluation of haemostasis [11,12]. Emerging studies suggest that ROTEM can identify hypercoagulability and fibrinolytic shutdown in septic patients, in some cases preceding abnormalities detected by standard SIC criteria and raising the concept of “impending SIC” [8,13,14,15]. Such findings highlight the potential role of viscoelastic testing in improving phenotypic characterization of coagulation disturbances in sepsis.

However, despite these promising insights, the clinical role of ROTEM in SIC remains incompletely defined. Current evidence is heterogeneous and largely observational, with variability in study design, patient populations, and interpretation of viscoelastic parameters. As a result, ROTEM is not yet established as a routine diagnostic tool for SIC, and its primary value appears to lie in risk stratification and haemostatic phenotyping rather than definitive diagnosis or treatment guidance [8,15,16].

Given the growing recognition of SIC as a key mediator of sepsis pathophysiology and the increasing use of viscoelastic assays in critical care, a comprehensive evaluation of the available evidence is warranted. This review aims to summarize current knowledge on the role of ROTEM in SIC, with a focus on its pathophysiological relevance, diagnostic performance, and potential implications for clinical decision-making.

## 2. Materials and Methods

This narrative review was based on a focused, non-systematic search of the PubMed database for articles addressing sepsis, sepsis-induced coagulopathy (SIC), disseminated intravascular coagulation (DIC), immunothrombosis, fibrinolysis, endothelial and platelet dysfunction, and viscoelastic testing, particularly rotational thromboelastometry (ROTEM). Additional relevant publications were identified through screening of reference lists from selected articles and review papers. Priority was given to clinically relevant studies, major reviews, and guideline or consensus documents in adult patients with sepsis or septic shock. The final selection of references was made on the basis of relevance to the pathophysiology, diagnosis, and potential clinical role of ROTEM in SIC.

## 3. Immunothrombosis in Sepsis: From PAMPs and DAMPs to Organ Failure

Host immune activation in sepsis arises from the combined sensing of pathogen-derived and host-derived danger signals. Both pathogen-associated molecular patterns (PAMPs) and damage-associated molecular patterns (DAMPs), released from injured or stressed cells, are recognized by pattern-recognition receptors (PRRs), including toll-like receptors (TLRs), expressed on immune and endothelial cells. This dual input—not PAMPs alone—drives the early dysregulated host response [17].

Activation of PRR signaling pathways induces the release of pro-inflammatory mediators such as TNF-α, IL-1β, IL-6, and interferons, while concurrently engaging the complement and coagulation systems [17]. DAMPs, including high mobility group box 1 (HMGB1), further amplify and sustain this response by perpetuating PRR activation even in the absence of ongoing infection. Persistent stimulation by both PAMPs and DAMPs contributes to endothelial injury, increased vascular permeability, and microcirculatory dysfunction [4,18].

This process evolves into a complex and paradoxical immune state characterised by simultaneous hyperinflammation and immunosuppression, ultimately leading to immune exhaustion and organ failure [17]. In parallel, neutrophil extracellular traps (NETs) released by activated neutrophils provide a scaffold that promotes platelet activation, aggregation, and contact pathway activation of coagulation, thereby enhancing intravascular thrombin generation and microvascular thrombosis [5]. Collectively, the sustained interaction between inflammatory, immune, and haemostatic pathways underpins the progression from a regulated host defense response to life-threatening organ dysfunction in sepsis.

## 4. Endothelial Dysfunction as a Central Driver of Sepsis Coagulopathy

The endothelium, a continuous monolayer of cells lining the vasculature, regulates vascular tone, coagulation, inflammation, and barrier integrity [5]. In sepsis, PAMPs, DAMPs, and inflammatory cytokines injure endothelial cells, triggering a shift to a pro-adhesive, pro-thrombotic, and pro-inflammatory phenotype, known as endothelial activation [19]. This leads to increased expression of adhesion molecules (e.g., ICAM-1, VCAM-1), promoting leukocyte adhesion and transmigration, and the loss of the glycocalyx, which compromises the semi-permeable barrier [20]. Activated endothelium downregulates anticoagulant proteins (thrombomodulin, endothelial protein C receptor) and releases procoagulant factors (von Willebrand factor, tissue factor), simultaneously promoting immunothrombosis—the pathological formation of microthrombi that obstruct microcirculation [5,21]. This endothelial injury and dysfunction are increasingly regarded as a central driver of organ failure in sepsis, directly linking the systemic inflammatory host response to the development of coagulopathy and subsequent multiple organ dysfunction [19].

## 5. Platelets as Immuno-Thrombotic Effectors in Sepsis

In sepsis, platelets act as key regulators of leukocyte function and inflammatory responses, linking immunity, inflammation, and thrombosis [22,23]. Activated by thrombin, PAMPs, and inflammatory cytokines, platelets interact with neutrophils and monocytes via P-selectin-PSGL-1 and other receptors, promoting leukocyte recruitment and modulating effector functions such as NET formation (NETosis) and phagocytosis [22,23]. NETosis can be induced through platelet–neutrophil contact and soluble mediators such as high-mobility group box 1 (HMGB1) [24]. Platelet-endothelial interactions further exacerbate endothelial dysfunction and loss of anticoagulant properties, reinforcing immunothrombotic responses in the microcirculation and contributing to multiple organ failure [23,25]. Furthermore, thrombocytopenia, reflecting increased platelet consumption and sequestration in the microvasculature, is a common biomarker of disease severity in sepsis [26]. While platelets contribute to early host defense through pathogen sequestration and leukocyte recruitment, this hyperactive state can progress to platelet exhaustion characterised by diminished responsiveness and a shift toward an anti-inflammatory phenotype [23]. Thus, platelets are critical, dual-function cells that drive both thrombotic and inflammatory pathways, making them central to the pathogenesis of sepsis-induced coagulopathy (SIC) [4,5,22,23].

## 6. Fibrinolytic Dysregulation and the Biphasic Nature of SIC

In sepsis, the fibrinolytic system undergoes profound dysregulation, transitioning from early fibrinolytic shutdown to a risk of subsequent hyperfibrinolysis, a dynamic process central to sepsis-induced coagulopathy (SIC) [6]. Early in sepsis, inflammatory mediators and endothelial activation promote a hypofibrinolytic phenotype, driven primarily by increased expression of plasminogen activator inhibitor-1 (PAI-1), which suppresses tissue plasminogen activator activity and limits plasmin generation [4,5].

This results in suppressed clot breakdown, contributing to persistent microvascular thrombi and exacerbating organ ischemia [27]. Endothelial injury, glycocalyx degradation, and platelet activation further reinforce antifibrinolytic signaling, integrating fibrinolytic suppression into the broader immunothrombotic response [21,28]. A subset of patients, particularly in severe inflammatory states, may exhibit hyperfibrinolysis, characterised by excessive release of tPA and neutrophil-derived proteases (e.g., elastase), which uncontrollably degrade fibrinogen and clots, leading to a consumptive coagulopathy [5,27]. Elevated PAI-1 levels and impaired clot lysis have been consistently associated with disease severity, disseminated intravascular coagulation, and mortality [4,29]. This biphasic dysfunction—early hypofibrinolysis promoting thrombosis and late hyperfibrinolysis risking bleeding—is critical to SIC pathology [4,6].

## 7. Sepsis-Induced Coagulopathy and Overt DIC: A Pathophysiological Continuum

Sepsis-induced coagulopathy (SIC) and disseminated intravascular coagulation (DIC) represent a continuum of coagulation disturbances in sepsis, driven by shared but progressively dysregulated pathophysiological mechanisms [6,7]. SIC is an early, predominantly prothrombotic state characterized by systemic thrombin generation, impaired endogenous anticoagulant pathways, and suppression of fibrinolysis, resulting in microvascular thrombosis and organ dysfunction [4,5]. This process is initiated by inflammatory mediators and pathogen- or damage-associated molecular patterns, which upregulate tissue factor expression on monocytes and endothelial cells, promote neutrophil extracellular trap formation, and induce endothelial activation, thereby amplifying thrombin generation and platelet-rich immunothrombi formation [4,18,24]. Concurrent downregulation of key anticoagulant pathways—including antithrombin, the protein C-thrombomodulin axis, and tissue factor pathway inhibitor—further shifts the haemostatic balance toward hypercoagulability [4,5].

As this dysregulated response progresses, persistent thrombin generation and fibrinolytic shutdown, mediated in part by increased plasminogen activator inhibitor-1, lead to widespread fibrin deposition and consumption of platelets and coagulation factors, marking the transition to overt DIC [7,29,30]. Unlike SIC, which is largely prothrombotic, DIC is characterized by a mixed thrombotic–haemorrhagic phenotype due to consumptive coagulopathy [7,30]. Clinically, SIC precedes DIC and can be identified using the ISTH SIC score, which incorporates platelet count, PT-INR, and SOFA score, with ≥4 indicating SIC [8,10]. Epidemiological data indicate that SIC occurs in approximately 24% of septic patients and up to 66% of those with septic shock, with mortality exceeding 30% [5,9,31]. SIC is independently associated with increased 28-day mortality and demonstrates a graded risk progression toward overt DIC [9,13,31,32]. In contrast, DIC is associated with more profound laboratory abnormalities—including thrombocytopenia, prolonged prothrombin time, elevated fibrin-related markers, and hypofibrinogenemia—and substantially higher mortality [7,33,34]. Diagnosis of overt DIC relies on validated scoring systems such as the ISTH criteria, which integrate platelet count, prothrombin time, fibrinogen level, and fibrin-related markers [30].

Importantly, bleeding risk is not significantly increased in SIC, distinguishing it from the later haemorrhagic manifestations of overt DIC [6,35]. This conceptual distinction underscores SIC as a critical therapeutic window, where early recognition using validated criteria may facilitate risk stratification and guide targeted anticoagulant or supportive interventions before progression to advanced coagulopathy [6,36,37].

## 8. Epidemiology and Prognostic Impact of SIC

Epidemiological data indicate that sepsis-induced coagulopathy (SIC), as defined by the ISTH SIC score, is both common and strongly associated with adverse outcomes in sepsis and septic shock [9,16,30]. Secondary analyses of large European randomized trials (HYPRESS and SISPCT) reported SIC prevalences of approximately 22–24% among patients with sepsis or septic shock, with SIC-positive patients exhibiting substantially higher 90-day mortality than SIC-negative patients (26.8% vs. 13.9% in HYPRESS) [9]. Single-country prospective cohorts have described higher prevalence figures; in one emergency department study, a SIC score ≥4 was present in 63.5% of patients with sepsis and septic shock and independently predicted 28-day mortality (odds ratio ~ 1.8) after adjustment for illness severity [30].

More recently, Schmoch et al. examined “impending SIC,” defined by near-threshold SIC scores, and showed that this intermediate group—although not meeting formal SIC criteria—already had significantly higher SOFA scores, increased need for organ support, and worse outcomes than stably SIC-negative patients, suggesting that progression along the SIC continuum begins before the conventional cutoff is reached [13]. Complementing these observations, Turcato et al. reported that patients with SIC had markedly higher risks of thrombosis, bleeding, and death compared with non-SIC sepsis patients in a prospective cohort, reinforcing SIC as a clinically relevant prothrombotic and prognostically unfavorable phenotype [31]. Across these studies, short-term mortality among SIC-positive or impending SIC patients commonly ranges from roughly 25% to more than 40%, consistently exceeding that of SIC-negative individuals [9,13,30,31]. Consequently, contemporary ISTH communications and narrative reviews regard SIC as an epidemiologically important, early sepsis-specific coagulopathy that identifies a high-risk subgroup and may serve to enrich populations for anticoagulant or adjunctive therapies in future interventional trials [4,16].

## 9. Laboratory Assessment of SIC: Conventional Coagulation Tests and SIC Scoring

In routine clinical practice, the assessment of sepsis-induced coagulopathy (SIC) relies on standard coagulation tests, prothrombin time (PT)/international normalized ratio (INR), fibrinogen, fibrin-related markers, and platelet count, which are widely available, rapidly obtainable, and amenable to serial monitoring [8,32]. These parameters form the basis for early identification of coagulation abnormalities in septic patients, although their sensitivity to dynamic haemostatic changes is limited.

The International Society on Thrombosis and Haemostasis (ISTH) DIC Subcommittee proposed a simplified SIC scoring system incorporating three variables: platelet count, PT or INR, and the Sequential Organ Failure Assessment (SOFA) score, thereby integrating coagulation abnormalities with the degree of organ dysfunction [38]. Platelet count is a central component of the score, with thresholds below 150 × 10^9^/L (1 point) and 100 × 10^9^/L (2 points), reflecting progressive consumption and disease severity [10]. PT-INR prolongation contributes additional points (1 point for INR > 1.2 and 2 points for INR > 1.4), indicating impaired coagulation factor activity [10].

In contrast to overt DIC scoring systems, the SIC score is designed to detect earlier and more subtle abnormalities, typically characterised by mild thrombocytopenia and modest PT prolongation in the context of evolving organ dysfunction [10,32]. Fibrinogen levels may remain within the normal range, limiting their discriminatory value at this stage. Consequently, the combination of platelet count and PT-INR, interpreted alongside SOFA, represents the cornerstone of bedside SIC screening and risk stratification.

Despite their clinical utility, conventional coagulation tests provide only a static and fragmented assessment of haemostasis and do not adequately capture clot dynamics or fibrinolytic activity. As such, they serve as a foundational framework upon which more advanced diagnostic modalities may build to improve phenotyping of septic coagulopathy [8,10,33].

## 10. Principles of Rotational Thromboelastometry (ROTEM)

Rotational thromboelastometry (ROTEM) is a whole-blood viscoelastic assay that provides a dynamic assessment of clot initiation, propagation, strength, and lysis, offering a more comprehensive evaluation of haemostasis than conventional plasma-based coagulation tests [11,12,34]. In ROTEM, citrated whole blood is placed in a cuvette containing specific activators (e.g., tissue factor for EXTEM, contact activator for INTEM, and platelet inhibitor for FIBTEM); a pin suspended in the sample rotates through a small angle while the cup remains stationary. As fibrin strands form and the clot stiffens, the viscoelastic coupling between blood and pin changes, generating a characteristic trace over time [11,12,34,35]. Standard ROTEM parameters include clotting time (CT), representing the time from test initiation to initial clot formation; clot formation time (CFT) and the alpha angle, which describe the kinetics of clot propagation; and maximum clot firmness (MCF), reflecting overall clot strength determined by fibrinogen concentration and platelet contribution [12,36]. Indices of fibrinolysis, such as maximum lysis (ML) or the lysis index at fixed time points (e.g., LI30 or LI60), provide quantitative information on clot stability and breakdown [12,36]. Unlike standard laboratory coagulation tests, which assess isolated components of the coagulation cascade in platelet-poor plasma, ROTEM evaluates the integrated function of coagulation factors, platelets, red blood cells, and fibrinolytic pathways in whole blood. This enables detection of both hypo- and hypercoagulable states, as well as fibrinolysis shutdown or hyperfibrinolysis [12,34,36]. Reviews of viscoelastic testing indicate that ROTEM parameters show moderate correlation with PT, aPTT, fibrinogen levels, and platelet count, while also reflecting the platelet contribution to clot strength and capturing dynamic fibrinolytic changes that may be missed by conventional assays [12,36]. In critical care and perioperative settings, ROTEM can be performed as a point-of-care test, with clinically actionable results typically available within 10–20 min. This supports goal-directed transfusion strategies and targeted use of coagulation factor concentrates, platelet transfusions, and antifibrinolytic or profibrinolytic therapies [34,35]. Collectively, these properties position ROTEM as a valuable adjunct to conventional coagulation testing for characterising complex, time-dependent haemostatic disturbances in critically ill patients, including those with sepsis-induced coagulopathy [8,34,35]. ROTEM is widely used in trauma and perioperative medicine, where it supports goal-directed haemostatic management and transfusion strategies [11,39,40]. In contrast, its use in general intensive care practice appears less widespread and is often concentrated in specialised centres, reflecting issues related to availability, standardisation, and clinical integration [8,34,40]. This gap is particularly relevant in sepsis, where current evidence is derived predominantly from observational and heterogeneous studies, underscoring the need for prospective research to define standardised ROTEM-based approaches and to better determine their impact on clinically meaningful outcomes [8,14,15,27].

## 11. ROTEM in Sepsis: Hemostatic Profiles and Prognostic Implications

The application of rotational thromboelastometry (ROTEM) in sepsis has revealed haemostatic patterns that complement conventional SIC scoring based on platelet count, PT/INR, and SOFA, particularly by capturing early hypercoagulability and fibrinolysis shutdown that may not yet be reflected in standard laboratory tests [8,14,27]. In a prospective cohort of patients with early sepsis and septic shock, Daudel and colleagues demonstrated that many patients exhibited increased clot firmness, especially in FIBTEM, together with prolonged clotting times and markedly elevated fibrinogen and D-dimer levels, consistent with an immunothrombotic, fibrinogen-driven hypercoagulable state rather than a simple consumptive coagulopathy [37]. More recently, a prospective ICU study of early sepsis found that, compared with reference values, sepsis patients had increased clot firmness mediated predominantly by fibrinogen, and that non-survivors showed subtly but significantly lower fibrinolytic activity than survivors, even though most ROTEM values remained within conventional reference ranges; a multivariable model incorporating ROTEM indices of the extrinsic pathway and fibrinolysis was among the strongest predictors of short-term mortality in that cohort [15]. The prognostic importance of fibrinolytic impairment was further underscored by an observational pilot study in septic shock, which showed that acute fibrinolysis shutdown—defined by high lysis indices on ROTEM—occurred early and was associated with higher morbidity and mortality in the studied population, with the degree of shutdown correlating with higher SOFA scores and non-survival [14]. These findings align with broader evidence that sepsis is characterised by hypofibrinolysis and microvascular thrombosis, and that functional assays such as ROTEM can help detect these alterations at the whole-blood level [27]. Within the SIC framework, such viscoelastic patterns have been interpreted as markers of “pre-SIC” or impending SIC, in which classical SIC criteria are not yet fulfilled, but ROTEM already reveals increased clot firmness and reduced fibrinolysis, which may help identify patients at increased risk of progression toward overt coagulopathy and organ failure and potentially suitable for targeted haemostatic interventions [8,14,15,27].

## 12. Methodological and Pathophysiological Limitations of ROTEM in Sepsis-Induced Coagulopathy

Despite its advantages in providing a dynamic, whole-blood assessment of coagulation, rotational thromboelastometry (ROTEM) has several important limitations in the evaluation of sepsis-induced coagulopathy (SIC). First, ROTEM is relatively insensitive to platelet dysfunction, as it predominantly reflects clot firmness driven by fibrinogen concentration and platelet count rather than qualitative platelet activity; consequently, impaired platelet function—common in sepsis due to inflammation, receptor downregulation, and circulating inhibitors—may be underestimated despite normal or elevated clot firmness parameters [12,22,23]. This limitation is particularly relevant in patients receiving antiplatelet therapy, where ROTEM may fail to detect pharmacologically induced platelet inhibition unless specific platelet function modules (e.g., ROTEM platelet) or mapping assays are employed, which are not routinely integrated into standard ROTEM protocols [12,34].

Second, ROTEM does not directly capture endothelial dysfunction, a central driver of SIC pathophysiology characterised by glycocalyx degradation, tissue factor expression, and dysregulated anticoagulant signaling; as a result, key processes such as endothelial activation and microvascular barrier disruption remain largely invisible to viscoelastic testing, despite their major contribution to thromboinflammation and organ failure [19,21]. Third, ROTEM provides limited insight into microvascular thrombosis, as it reflects global haemostatic potential in a macroscopic sample rather than localized intravascular clot formation, which is particularly relevant in SIC where microthrombi formation in the capillary bed may occur despite relatively preserved or even hypercoagulable ROTEM profiles [7,25]. Finally, although ROTEM can detect fibrinolytic shutdown, it may not fully differentiate between impaired fibrinolysis and reduced clot breakdown secondary to other factors, limiting its specificity in complex septic states [14]. Collectively, these limitations highlight that ROTEM should be interpreted in conjunction with conventional laboratory tests and the overall clinical context, rather than as a standalone diagnostic tool, when assessing coagulation abnormalities in SIC (Table 1).

## 13. Therapeutic Implications: Anticoagulation Strategies in SIC and DIC

Therapeutic management of sepsis-induced coagulopathy (SIC) rests on two pillars: optimal treatment of the underlying infection and organ support, and selective use of anticoagulant strategies in carefully chosen patients based on SIC or DIC criteria [8,16]. Current international guidance emphasizes that broad, unselected anticoagulation for all patients with sepsis is not supported by the available evidence and may increase bleeding risk; instead, anticoagulant therapy should be considered primarily in those with SIC or sepsis-associated DIC and low bleeding risk [8,16,38]. The ISTH SSC communication on SIC highlights unfractionated or low-molecular-weight heparin (LMWH), antithrombin concentrates, and recombinant human soluble thrombomodulin (rhTM) as principal candidates, while stressing that none has yet demonstrated unequivocal survival benefit in adequately powered randomized trials [8,38].

A systematic overview of randomized trials and meta-analyses found that anticoagulant therapy in sepsis can improve DIC resolution rates—particularly with antithrombin and rhTM—but has not consistently reduced overall mortality, likely due to heterogeneous populations, late treatment, and inclusion of patients without overt coagulopathy [39,40,42]. Observational data from Japanese registries suggest that antithrombin supplementation and rhTM, alone or in combination, may confer survival benefit in sepsis-induced DIC without a major increase in serious bleeding, especially when initiated early and guided by DIC scores; however, these findings remain hypothesis-generating [8,40,43]. Recent systematic reviews of anticoagulant therapy in sepsis therefore conclude that anticoagulants should not be used routinely but may be reasonable in selected high-risk phenotypes—such as patients fulfilling SIC or overt DIC criteria with high illness severity-ideally within clinical trials or under rigorous monitoring [16,38,39,40]. Future research priorities include refining SIC-based enrichment strategies, integrating biomarkers of fibrinolysis and endothelial injury to identify anticoagulant-responsive subgroups, and testing combination approaches that pair anticoagulation with anti-inflammatory and endothelial-stabilizing therapies [8,38,39,40,44].

## 14. Conclusions—Positioning ROTEM Within SIC Management

Sepsis is now understood as life-threatening organ dysfunction caused by a dysregulated host response to infection, in which the immune, endothelial, and haemostatic systems interact to drive microvascular thrombosis, organ failure, and high short- and long-term mortality. Large contemporary epidemiological analyses indicate that sepsis accounts for over 160 million incident cases and more than 20 million deaths annually, with the greatest burden borne by older adults. Within this syndrome, sepsis-induced coagulopathy (SIC) represents an early, predominantly prothrombotic haemostatic disturbance characterised by systemic thrombin generation, impaired endogenous anticoagulant pathways, and fibrinolytic suppression, often progressing along a continuum toward overt disseminated intravascular coagulation and further worsening outcomes. The ISTH SIC score, based on platelet count, PT/INR, and SOFA, operationalizes this concept at the bedside and consistently identifies a subgroup of septic patients at increased risk of mortality, despite the absence of overt bleeding, underscoring SIC as a clinically relevant procoagulant phenotype.

Rotational thromboelastometry and related viscoelastic assays complement conventional laboratory tests by providing a dynamic assessment of clot formation and fibrinolysis. Available evidence suggests that ROTEM frequently demonstrates fibrinogen-driven hypercoagulability and reduced fibrinolysis in septic patients, patterns that may precede conventional SIC criteria. However, these observations are derived primarily from observational and heterogeneous studies, and their role in routine diagnosis remains uncertain. At present, the main clinical value of ROTEM appears to lie in risk stratification and phenotyping, enabling identification of distinct haemostatic profiles that may be associated with disease severity and outcomes rather than serving as a standalone diagnostic tool for SIC.

Therapeutically, current guidance prioritizes prompt infection control and organ support while reserving anticoagulant strategies—such as heparins, antithrombin concentrates, and recombinant thrombomodulin—for carefully selected patients with SIC or sepsis-associated DIC and low bleeding risk, as randomized trials have not demonstrated consistent survival benefit in unselected sepsis populations. Collectively, these insights support a role for SIC-based stratification in clinical research and highlight the potential of viscoelastic testing to refine patient phenotyping. Nevertheless, further prospective studies are required to establish standardized thresholds and to determine whether ROTEM-guided approaches can improve clinical outcomes in septic coagulopathy.

## Figures and Tables

**Table 1:** Summary of clinical evidence on ROTEM in SIC.

Population/Setting	Main ROTEM Findings	Relation to SIC/Outcomes	Study (Year)
30 patients with septic shock (observational pilot study)	High lysis indices indicating acute fibrinolysis shutdown occurred early; shutdown associated with higher SOFA scores and non-survival	Linked ROTEM-defined fibrinolysis shutdown to increased morbidity and mortality; supports hypofibrinolysis as a key SIC feature	Schmitt et al. 2019 [14]
50 ICU patients with early sepsis (prospective cohort)	Increased MCF, largely fibrinogen-mediated; subtle but significantly lower fibrinolytic activity in non-survivors despite values often within reference ranges	ROTEM indices of extrinsic pathway and fibrinolysis improved prediction of short-term mortality; supports use of ROTEM for risk stratification in early SIC	Czempik & Wiórek 2024 [15]
30 adults with early and established sepsis in ICU (prospective cohort)	Increased clot firmness (especially FIBTEM), prolonged CT, elevated fibrinogen and D-dimer; pattern consistent with fibrinogen-driven hypercoagulability rather than simple consumption	Demonstrated early hypercoagulable ROTEM profile in sepsis; suggested immunothrombotic state that may precede overt SIC/DIC and relate to organ dysfunction	Daudel et al. 2009 [41]

## Data Availability

No new data were created or analyzed in this study.

## Abbreviations

aPTT	Activated partial thromboplastin time
AT	Antithrombin
CT	Clotting time (ROTEM parameter)
CFT	Clot formation time (ROTEM parameter)
DAMPs	Damage-associated molecular patterns
DIC	Disseminated intravascular coagulation
FDP	Fibrin degradation products
HMGB1	High-mobility group box 1
ICAM-1	Intercellular adhesion molecule-1
ICU	Intensive care unit
INR	International normalized ratio
ISTH	International Society on Thrombosis and Haemostasis
LMWH	Low-molecular-weight heparin
MCF	Maximum clot firmness (ROTEM parameter)
ML	Maximum lysis (ROTEM parameter)
NETs	Neutrophil extracellular traps
PAMPs	Pathogen-associated molecular patterns
PAI-1	Plasminogen activator inhibitor-1
PRRs	Pattern-recognition receptors
PT	Prothrombin time
PT-INR	Prothrombin time–international normalized ratio
ROTEM	Rotational thromboelastometry
SIC	Sepsis-induced coagulopathy
SOFA	Sequential Organ Failure Assessment
SSC	Scientific and Standardization Committee
tPA	Tissue plasminogen activator
TEG	Thromboelastography
TF	Tissue factor
TLR	Toll-like receptor
TNF-α	Tumour necrosis factor alpha
VCAM-1	Vascular cell adhesion molecule-1
